# WNT5A promotes stemness characteristics in nasopharyngeal carcinoma cells leading to metastasis and tumorigenesis

**DOI:** 10.18632/oncotarget.3518

**Published:** 2015-03-10

**Authors:** Li Qin, Yan-Tao Yin, Fang-Jing Zheng, Li-Xia Peng, Chang-Fu Yang, Ying-Na Bao, Ying-Ying Liang, Xin-Jian Li, Yan-Qun Xiang, Rui Sun, An-Hua Li, Ru-Hai Zou, Xiao-Qing Pei, Bi-Jun Huang, Tie-Bang Kang, Duan-Fang Liao, Yi-Xin Zeng, Bart O. Williams, Chao-Nan Qian

**Affiliations:** ^1^ Sun Yat-sen University Cancer Center, State Key Laboratory of Oncology in South China, Collaborative Innovation Center for Cancer Medicine, Guangzhou, China; ^2^ Hunan Province Engineering Research Center of Bioactive Substance Discovery of Traditional Chinese Medicine, School of Pharmacy, Hunan University of Chinese Medicine, Changsha, Hunan, China; ^3^ Division of Pharmacoproteomics, Institute of Pharmacy and Pharmacology, University of South China, Hengyang, Hunan, China; ^4^ Department of Radiotherapy, Ningde Municipal Hospital, Fujian Medical University Affiliated Hospital, Ningde, Fujian, China; ^5^ Brain Tumor Center and Department of Neuro-Oncology, The University of Texas MD Anderson Cancer Center, Houston, Texas, USA; ^6^ Department of Nasopharyngeal Carcinoma, Sun Yat-sen University Cancer Center, Guangzhou, Guangdong, China; ^7^ Department of Ultrasonography, Sun Yat-sen University Cancer Center, Guangzhou, Guangdong, China; ^8^ Division of Stem Cell Regulation and Application, School of Pharmacy, Hunan University of Chinese Medicine, Changsha, Hunan, China; ^9^ Laboratory of Cell Signaling and Carcinogenesis, Van Andel Research Institute, Grand Rapids, Michigan, USA

**Keywords:** nasopharyngeal carcinoma, WNT5A, metastasis, tumorigenesis, PKC

## Abstract

Nasopharyngeal carcinoma (NPC) has the highest metastasis rate among head and neck cancers with unclear mechanism. WNT5A belongs to the WNT family of cysteine-rich secreted glycoproteins. Our previous high-throughput gene expression profiling revealed that WNT5A was up-regulated in highly metastatic cells. In the present study, we first confirmed the elevated expression of WNT5A in metastatic NPC tissues at both the mRNA and protein levels. We then found that WNT5A promoted epithelial-mesenchymal transition (EMT) in NPC cells, induced the accumulation of CD24-CD44+ cells and side population, which are believed to be cancer stem cell characteristics. Moreover, WNT5A promoted the migration and invasion of NPC cells in vitro, while in vivo treatment with recombinant WNT5A promoted lung metastasis. Knocking down WNT5A diminished NPC tumorigenesis in vivo. When elevated expression of WNT5A coincided with the elevated expression of vimentin in the primary NPC, the patients had a poorer prognosis. Among major signaling pathways, protein kinase C (PKC) signaling was activated by WNT5A in NPC cells. A positive feedback loop between WNT5A and phospho-PKC to promote EMT was also revealed. Taken together, these data suggest that WNT5A is an important molecule in promoting stem cell characteristics in NPC, leading to tumorigenesis and metastasis.

## INTRODUCTION

Nasopharyngeal carcinoma (NPC) is a prevalent malignancy in South China and Southeast Asia [1-4]. It is sensitive to radiotherapy and radiochemotherapy; however, approximately 30% of NPC patients will develop distant metastases [5]. Distant metastasis has been the main reason for treatment failure, though the underlying mechanism(s) has not been fully elucidated. Using our established NPC-metastasis cellular and animal models [6, 7], we have previously performed high-throughput gene expression profiling followed by functional studies to identify and validate the key molecules responsible for promoting NPC metastasis, including serglycin, interleukin-8, and HSP27 [7-9]. Interestingly, *WNT5A* was one of the genes found to be over-expressed in the high-metastasis NPC cells both *in vitro* and *in vivo* [7]. However, its clinical relevance and its real functions in NPC development are undetermined.

WNT5A belongs to the large WNT family of cysteine-rich secreted glycoproteins, which includes at least 19 members in humans [10, 11]. In normal cells, WNT proteins control cell fate, migration, and cellular polarity through cell surface receptors that modulate the transcription of specific target genes. Recently, WNT5A was found to be a critical molecule regulating the migration of stem cells during embryonic development [12], as well as the proliferation and repopulation of hematopoietic stem cells [13]. WNT5A signaling has been classified as a non-canonical and non-transforming pathway [14]. Based on results obtained in both *Xenopus* and mammalian cells, the biological effects of WNT5A are known to depend on the Wnt/Ca^2+^ pathway. For example, Wnt5a can signal through frizzled receptor (Fz) 5 and thereby activate protein kinase C (PKC) in malignant melanomas [15, 16].

The role of WNT5A in tumorigenesis remains ambiguous. In cellular and animal models of hematopoietic malignancies [17], colorectal cancer [18], thyroid carcinoma [19], and breast cancer [20], WNT5A has been shown to inhibit tumor cell proliferation and invasion. The loss of one *WNT5a* allele in a mouse model is associated with the occurrence of hematopoietic malignancies [17]. WNT5A overexpression can suppress the expression of the metastasis suppressor Kiss-1 [15]. There is also evidence that increased WNT5A expression is associated with cancer progression [21] and with the movement and invasiveness of melanoma cells [16]. Up-regulation of WNT5A has also been reported in cancers of the lung, breast, and stomach [22-24]. The essential roles of WNT5A in macrophage-induced cancer invasiveness is also reported [25].

In the present study, we aimed to explore the roles of WNT5A in the stemness characteristics of NPC cells responsible for NPC metastasis.

## RESULTS

### Up-regulation of WNT5A is associated with NPC metastases in clinical scenarios

**Figure 1:**
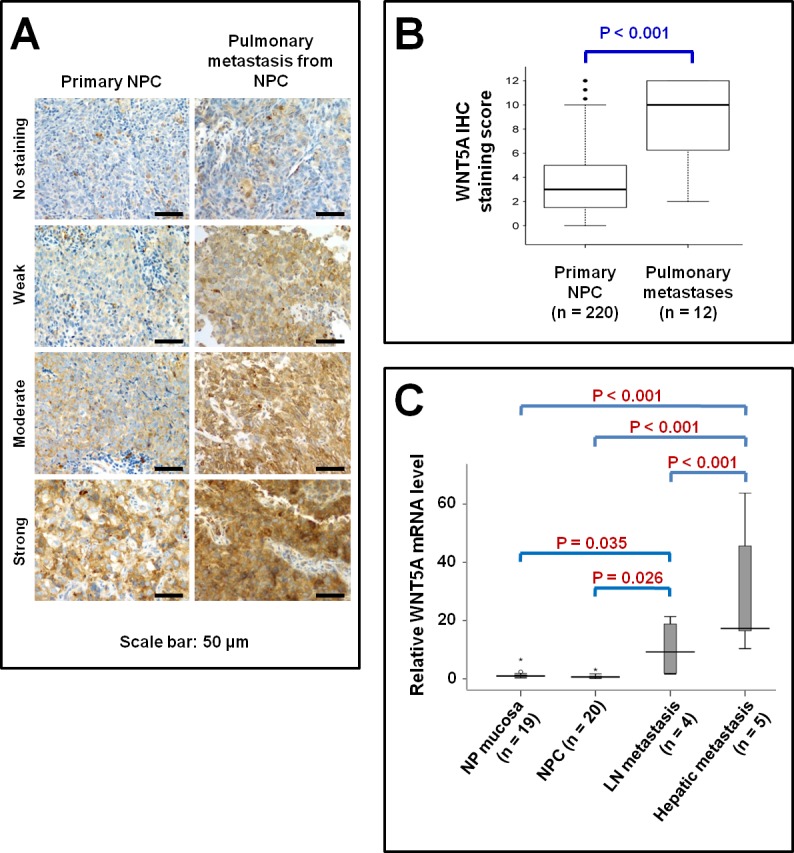
Elevated WNT5A expression in metastatic NPC tissues A, IHC staining of WNT5A in primary NPC tissues as well as in pulmonary metastatic NPC tissues. B, WNT5A mRNA expression in the pulmonary metastatic NPC tissues. C, The relative levels of *WNT5A* mRNA expression in different human tissues measured using quantitative real time-PCR. Fold change (y-axis) represents the relative expression of the gene in different cancerous tissues compared with the level of *WNT5A* mRNA expression in nasopharyngeal (NP) mucosa, normalized to GAPDH gene expression. The highest *WNT5A* mRNA level was found in the hepatic metastasis, followed by lymph node metastasis.

Cancer stem cells have been reported to be responsible for the aggressiveness and metastasis of different malignancies [26-28]. We therefore detected the level of *WNT5A* expression in metastatic NPC tissues (Figure 1). WNT5A protein was highly expressed in pulmonary metastases from NPC, and the *WNT5A* mRNA level was also elevated in hepatic metastases from NPC. These findings were consistent with our previous findings that *WNT5A* mRNA was overexpressed in high-metastasis NPC S18 cells [7]. These data collectively showed a close correlation between WNT5A expression level and NPC cell metastasis, implying an important role for WNT5A in NPC progression.

### WNT5A promotes the migration, invasion, and metastasis of NPC cells

**Figure 2:**
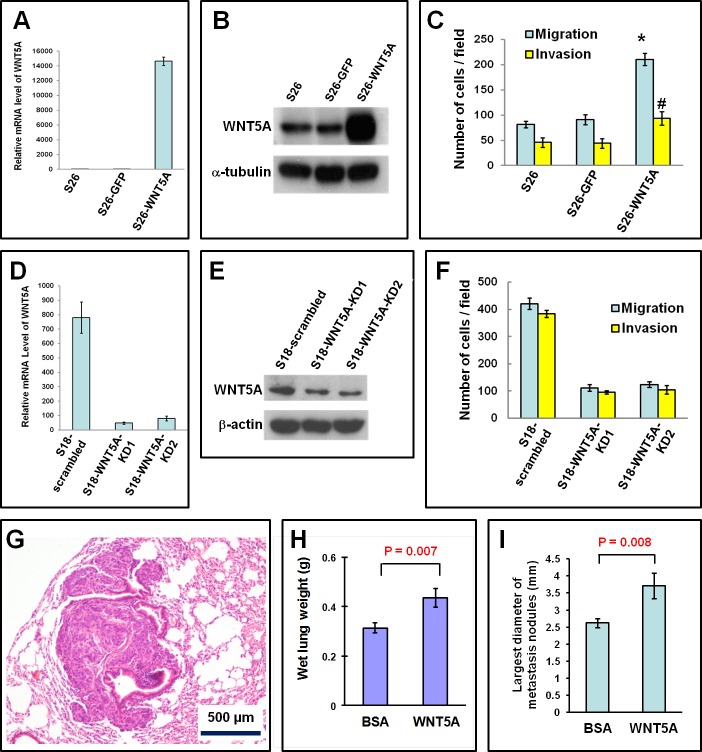
WNT5A promotes migration, invasion, and metastasis of NPC cells A and B, Overexpression of both WNT5A mRNA (A) and protein (B) was achieved in low-metastasis S26 cells by transfection of a *WNT5A* expression vector. C, The migration and invasion of S26 cells was significantly promoted by WNT5A overexpression. D and E, Knocking down WNT5A in S18 cells resulted in reduced *WNT5A* mRNA (D) and protein (E). F, The migration and invasion of S18 cells was significantly inhibited by knocking down WNT5A. G, Histological image of a lung metastasis in a nude mouse after tail vein injection of S26 cells. H, The wet lung weights of the mice treated with rWNT5A were significantly higher, suggesting more metastases into the lung. I, The largest diameters of the pulmonary metastatic nodules in the mice treated with rWNT5A were also significantly larger, suggesting that rWNT5A promoted the metastasis of NPC cells.

We further explored whether overexpression of WNT5A could promote the motility and metastasis of NPC cells. Overexpression of WNT5A in S26 cells significantly promoted migration and invasion (Figure 2A-2C). In contrast, stable knock-down of WNT5A in S18 cells significantly inhibited migration and invasion (Figure 2D-2F). *In vivo* animal experiments showed that administration of recombinant WNT5A protein significantly promoted lung metastasis (Figure 2G-2I). Together, these findings confirmed that WNT5A promoted the motility and metastatic ability of NPC cells, which are typical characteristics of cancer stem cells.

### Tumorigenesis of NPC cells depends on the expression of WNT5A

Tumorigenesis is a hallmark of cancer stem cells. By evaluating tumorigenesis in S18 cells, we surprisingly found that tumorigenesis was almost completely abolished after knocking down WNT5A (Table 1). This finding suggested that WNT5A had an essential role in tumorigenesis in NPC cells.

**Table 1:** Tumorigenesis of S18 cells after stable knocking-down of WNT5A gene

Number of S18 cells injected	Number of tumors formed in nude mice		
	Scrambled	WNT5A-KD1	WNT5A-KD2
1×10^5^	5/5	0/5	0/5
1×10^6^	5/5	0/5	0/5
1×10^7^	5/5	0/5	1/5

### WNT5A up-regulates stem-like cell markers in NPC cells

**Figure 3:**
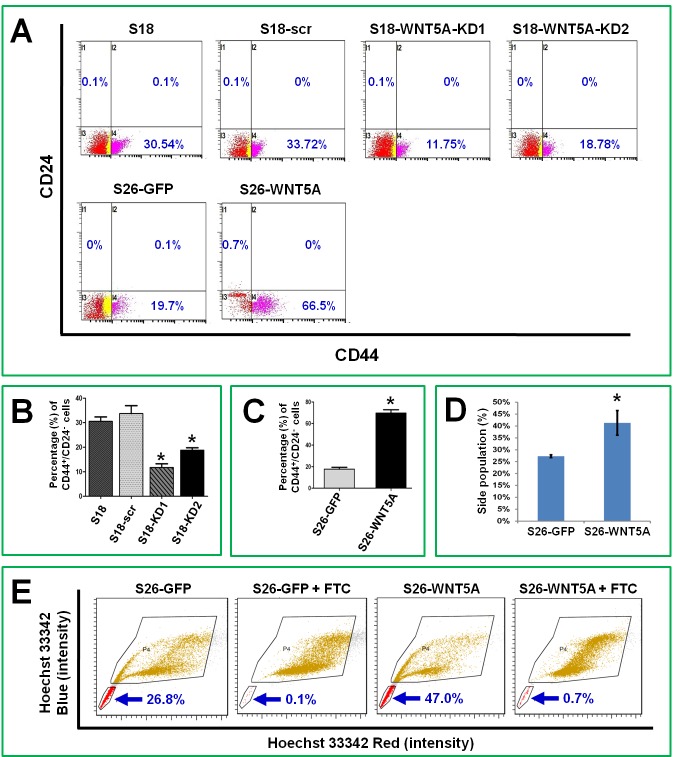
WNT5A regulates stem-like cell markers in NPC cells A, Flow cytometry analyses showed that stable knocking down WNT5A in S18 cells could significantly decrease the percentage of CD24-/CD44+ cells, which are believed to possess stemness characteristics. Over-expression of WNT5A in S26 cells by transfecting expression vector could significantly increase the percentage of CD24-/CD44+ cells. B and C, Comparisons of the detected values from the experiments shown in A. D, Side population analyses showed that over expression of WNT5A in S26 cells resulted in significant increase of the side population. E, the images of flow cytometry for side population analysis. FTC, fumitremorgin C, an inhibitor of ABCG2 protein. *, *P* < 0.01.

The immunophenotype CD44+CD24- has been used to identify cancer stem-like cells in head and neck cancers [29-31]. We performed flow cytometry analyses to measure the percentage of CD44+CD24- cells after knocking down or over-expressing WNT5A (Figure 3A-3C). Knocking down WNT5A in the high-metastasis S18 cell line resulted in a significant reduction in the percentage of stem-like cells, and overexpression of WNT5A increased the percentage of stem-like cells in the low-metastasis line. Accumulation of side population (defined as cells that show higher efflux of DNA-binding dye Hoechst 33342) has also been reported to be one of the properties of cancer stem cells. We found that over-expression of WNT5A in S26 cells could significantly increase the percentage of side population (Figure 3D and 3F). These findings confirmed that WNT5A regulated the stem cell characteristics of NPC cells.

### WNT5A promotes EMT and up-regulates vimentin protein levels, while co-overexpression of WNT5A and vimentin predicts poorer survival

**Figure 4:**
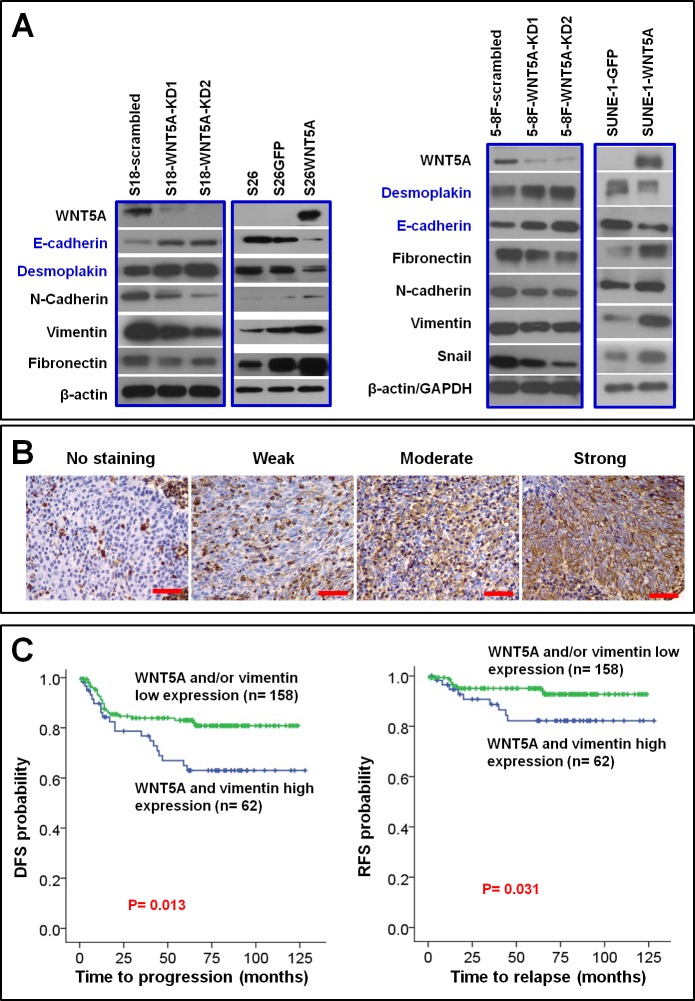
WNT5A promotes EMT in NPC cells, and this procedure associates with poor patient survival A, Knocking down WNT5A in high-metastasis S18 and 5-8F cells resulted in reduction of mesenchymal proteins vimentin, N-cadherin, and fibronectin, as well as increment of epithelial protein desmoplakin and E-cadherin, whereas over-expression of WNT5A in the low-metastasis S26 and SUNE-1 cells resulted in accumulation of mesenchymal proteins and reduction of epithelial proteins. B, IHC staining of vimentin in NPC tissue. C, By combining the IHC scores of vimentin shown in B with the WNT5A IHC score shown in Figure 1A, survival analyses were performed in a cohort of 220 patients. Shorter disease-free survival (DFS) and relapse-free survival (RFS) were observed in the patients with primary NPC expressing high levels of both WNT5A and vimentin proteins.

Epithelial-mesenchymal transition (EMT) generates cells with stem cell properties [32, 33]. Vimentin is a mesenchymal stem cell marker [34, 35] that is usually up-regulated in EMT of NPC cells [9, 36]. Moreover, vimentin has been reported to be positively correlated with clinically more aggressive NPC behaviors [37]. In the present study, we found that WNT5A positively regulated the level of vimentin among the other markers of EMT in NPC cells (Figure 4A). We further explored the clinical implications of co-expression of WNT5A and vimentin using IHC staining in TMA of NPC tissues (Figure 4B). Interestingly, we found that co-overexpression of WNT5A and vimentin in primary tumors was significantly correlated with decreased disease-free survival and relapse-free survival in NPC patients (Table 2 and Figure 4C).

**Table 2:** Association between co-overexpression of WNT5A plus vimentin and clinical characteristics in 220 patients with low differentiated NPC

Characteristics	No.	WNT5A plus vimentin elevated expression		P value (Chi-square test)
		Yes	No	
Gender				
Male	170	46	124	0.495
Female	50	16	34	
Age				
< 46	113	36	77	0.213
≥ 46	107	26	81	
T stage				
T1-2	84	23	61	0.836
T3-4	136	39	97	
N stage				
N0-1	138	33	105	0.068
N2-3	82	29	53	
Clinical staging				
I - II	55	12	43	0.226
III – IV	165	50	115	
Death				
Yes	86	26	60	0.588
No	134	36	98	
Disease progression				
Yes	46	20	26	**0.010**
No	174	42	132	
Distant metastasis				
Yes	29	11	18	0.210
No	191	51	140	
Locoregional recurrence				
Yes	18	9	9	**0.032**
No	202	53	149	

Note: Disease progression is defined by locoretional recurrence and/or distant metastasis.

### A positive feedback loop between WNT5A and phosphorylated PKC regulates EMT in NPC cells

**Figure 5:**
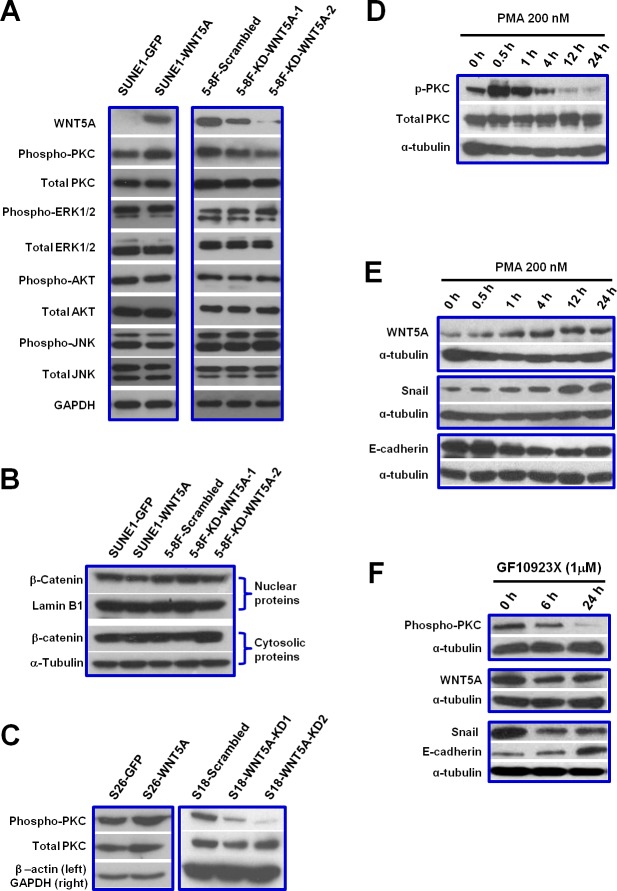
WNT5A activates PKC signaling A positive loop between WNT5A and phospho-PKC promotes EMT in NPC cells. A, By using SUNE-1 cells with WNT5A over-expression as well as 5-8F cells with WNT5A knocked-down, several major signaling pathways were examined, including PKC, ERK, AKT, and JNK pathways. Only phosphorylation of PKC was altered by WNT5A expression in the cells. B, Over-expression or knocking down of WNT5A did not alter the protein level of β-catenin in the nucleus, indicating that the classic β-catenin signaling pathway could not be activated by WNT5A in NPC cells. C, Over-expression of WNT5A increased phospho-PKC level, while knocking down WNT5A resulted in reduction of phosphorylated PKC. D, PKC activator PMA could successfully induce phosphorylation of PKC in S26 cells. E, Activation of PKC by PMA in S26 cells could further up-regulate WNT5A level and subsequently increase mesenchymal marker Snail and decrease epithelial marker E-cadherin. F, PKC inhibitor GF10923X could decrease WNT5A level in S18 cells and subsequently reduce Snail and gain E-cadherin.

**Figure 6:**
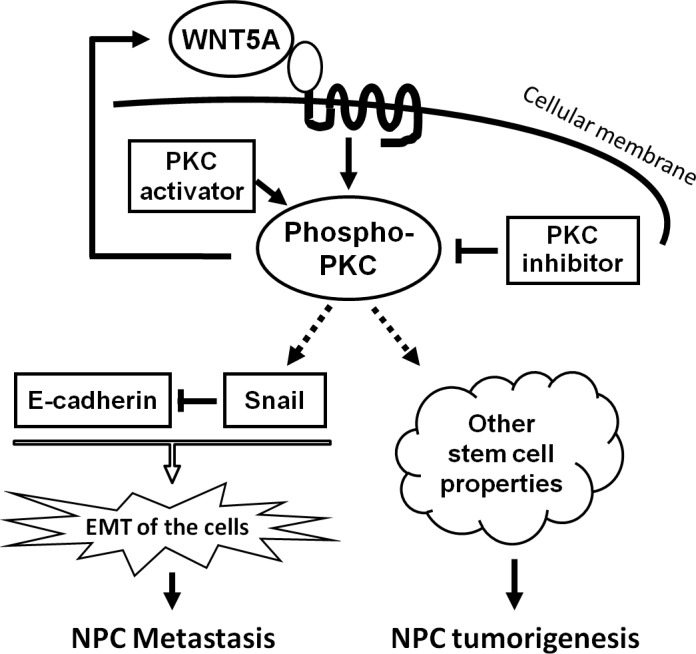
A schematic model to summarize WNT5A signaling in NPC WNT5A activates PKC signaling, subsequently promotes EMT and the presentation of other stem cell properties, resulting in NPC metastasis and tumorigenesis. A positive loop between WNT5A and phospho-PKC is an ideal therapeutic target.

We further examined the roles of WNT5A in several major signaling pathways underlying tumor progression, including PKC, ERK, AKT, and JNK pathways. PKC signaling was the one activated by WNT5A in NPC cells (Figure 5A). However, the classical β-catenin signaling was not activated by WNT5A (Figure 5B). We further tested whether the positive feedback loop between WNT5A and phospho-PKC could regulate EMT in NPC cells. First, we found that stably knocking down *WNT5A* mRNA using shRNAs in high-metastasis S18 cells significantly diminished PKC phosphorylation, and overexpression of WNT5A up-regulated phospho-PKC in low-metastasis S26 cells (Figure 5C). Activating PKC using the PKC activator PMA in S26 cells (Figure 5D) induced the accumulation of WNT5A and subsequently induced the up-regulation of the mesenchymal marker Snail and a reduction in the epithelial marker E-cadherin (Figure 5E). Inhibiting PKC phosphorylation in S18 cells using GF10923X reduced the phospho-PKC level and down-regulated the WNT5A level with a consequent reduction in Snail and an increase in E-cadherin (Figure 5F). Taken together, the positive feedback loop between phospho-PKC and WNT5A triggered EMT in NPC cells (Figure 6).

## DISCUSSION

In contrast to other squamous cell carcinomas of the head and neck, NPC is characterized by a high tendency to metastasize. Between 5% and 8% of NPC patients present with distant metastases at diagnosis [38, 39], and 30% of patients with stage III or IV disease will develop distant metastases after standard treatment [40]. Gaining stem cell characteristics in a portion of NPC cells has been proposed to be an important factor responsible for systemic spreading and treatment resistance of this fetal disease [41].

In the present study, we demonstrated that WNT5A enhanced the stem cell properties of NPC cells, promoted tumorigenesis, migration, invasion, and metastasis in NPC cells, regulated vimentin expression, and correlated with a poorer patient prognosis. Moreover, the elevated WNT5A expression levels in metastatic NPC tissues suggested the clinical relevance of WNT5A in NPC metastasis. A positive feedback loop between WNT5A and phospho-PKC in regulating EMT was also revealed, suggesting that breaking this loop could be a useful strategy in NPC treatment. Our findings are consistent with the findings in breast cancer, in which a positive feedback loop has also been reported between WNT5A and PKC activation [42].

Emerging evidence shows that carcinoma cells activate the dormant EMT program in promoting cell migration, invasion, and metastasis [43, 44], and EMT is believed to be a crucial step in the conversion of early-stage disease to invasive and metastatic cancer. The findings in the present study implied that WNT5A-driven EMT might be the cause of poorer patient survival in NPC patients because the co-elevated expression of WNT5A and vimentin correlated with shorter disease-free and relapse-free survival times. Our findings are consistent with a recent report that WNT5A can promote the stem cell-like properties of breast cancer cells [37].

In summary, we found that WNT5A played a strong role in regulating NPC tumorigenesis and metastasis via activating phospho-PKC, EMT, and the stemness characteristics of NPC cells. A positive feedback loop between WNT5A and phospho-PKC was also revealed. Therefore, both PKC and WNT5A are key molecules in NPC metastasis and are potential therapeutic targets for its prevention and treatment.

## MATERIALS AND METHODS

### Cell lines and clones

The NPC line CNE-2 and its clones S18, S22, and S26 have previously been established and reported [6]. S18 has the highest metastatic ability; S22, S26, and the parental line CNE-2 have low metastatic ability. Another NPC cell line SUNE-1 (low-metastasis ability) and its high-metastasis clone 5-8F [7] were also used. All of the cells were cultured in Dulbecco's modified Eagle's medium (DMEM) with 10% fetal bovine serum purchased from Gibco/BRL (Grand Island, NY, USA), 100 units/ml penicillin G, 100 units of streptomycin, and 2 mmol/L L-glutamine (all from Invitrogen, Carlsbad, CA, USA). Cells were incubated at 37°C in a humidified atmosphere containing 5% CO_2_.

### Stable knockdown of WNT5A in high-metastasis S18 cells

S18 cells stably expressing either WNT5A shRNA or a scrambled non-target shRNA were established using a BLOCK-iT™ Lentiviral Pol II miR RNAi system (Invitrogen, Carlsbad, CA) according to the manufacturer's instructions. Scrambled plasmid: SHC002 (Sigma), mission shRNA plasmid DNA (target: NM_003392.3) (Sigma): TRCN0000296083 and TRCN0000296137.

The following are target sequences of WNT5A mission shRNA plasmid DNA (Sigma):

Sequence 1 (for S18-WNT5A-sh1 cells): 5′-TCCCAGGACCCGCTTATTTA-3′;
Sequence 2 (for S18-WNT5A-sh2 cells): 5′-AAAGAATGCCAGTATCAATT-3′.

Lentiviruses were produced by cotransfecting 293FT cells with one of the expression plasmids and the MISSION Lentiviral Packaging Mix SHP001-1.7 ml (Sigma) using Lipofectamine 2000 (Invitrogen, Carlsbad, CA) as a transfection reagent. Infectious lentiviruses were harvested 72 h after transfection, centrifuged to remove cell debris, and filtered through 0.45-μm filter (Millipore, Bedford, MA). Cells were transduced with lentiviruses expressing WNT5A or scrambled non-target shRNAs and then cultured in medium containing 5 μg/ml blasticidin (Sigma) for 21 days to eliminate the untransduced cells. Quantitative PCR and immunoblotting for WNT5A were performed to determine the knockdown efficiency.

### Stable overexpression of WNT5A in low-metastasis S26 cells

Lentiviral particles were packaged and used for cell transduction according to the manufacturer's instructions (Invitrogen). The Clone-26 cells (30-50% confluency) were transfected with pLenti6/V5-Wnt5a or pLenti6/V5 (Invitrogen, San Diego) using Lipofectamine 2000 (Invitrogen) according to the manufacturer's protocol. After incubation for 24 h, the selection reagent blasticidin (5 μg/mL; Invitrogen, San Diego) was added to select stably transfected clones. Selection was continued for 21 days, with the medium being refreshed every 3 days. Cells transfected with vector pLenti6/V5-Wnt5a were called S26Wnt5a. Cells transfected with vector pLenti6/V5 were named as S26GFP.

### Tumorigenesis and treatment with rWNT5A *in vivo*

Female BALB/c (nu/nu) nude mice at 4-6 weeks of age were used. For the tumorigenesis experiments, cancer cells at different concentrations in 100 μl DMEM were injected subcutaneously into the flank of each mouse. Three weeks after inoculation, the tumors were observed and recorded, and the mice were euthanized. For the treatment of rWNT5 (R&D System), 48 mice were randomized into two groups. S26 cells were pre-treated with 0.05 μg/mL rWnt5A protein (for the experimental group) or bovine serum albumin (BSA) (for the control group) for 16 h followed by tail vein injection (5 × 10^5^ cells in 100 l of cell suspension per mouse). After tumor cell injection, the experimental group received intraperitoneal injection of rWNT5A (75 ng of rWNT5A dissolved in 100 μl of 0.1% BSA per mouse, which is an effective dose to induce melanoma cell metastasis [33]) on days 3, 6, 9, 12, 15, 18. The control group received intraperitoneal injections of 100 μl of 0.1% BSA per mouse on the same time schedule. The experiment was terminated 6 weeks after the first inoculation. After euthanasia, the lungs of each mouse were isolated intact, and the wet lung weight was measured. The largest diameter of the metastatic nodules in each mouse was also measured. The lungs were then fixed in 4% paraformaldehyde in PBS overnight, embedded in paraffin, and processed for routine histological H&E staining.

### Transfection with siRNAs against WNT5A and treatments with recombinant WNT5A, PKC inhibitor, and PKC activator *in vitro*

Three siRNAs for *WNT5A* (Ruibo Biotechnology Company, Guangzhou, China) were transfected into cells (30-50% confluency) using Lipofectamine^TM^ RNAi MAX reagent (Invitrogen, Carlsbad, CA) according to the manufacturer's instructions. For recombinant WNT5A (rWNT5A; R&D systems, Minneapolis, MN) treatment, the rWNT5A was reconstituted in sterile PBS containing 0.1% BSA to a stock concentration of 10 μg/ml. Based on the literature [15], we chose a concentration of 0.2 μg/ml and 12 h as the dose and time points.

For protein kinase C (PKC) inhibition studies, GF-10923X (an inhibitor of PKCα, β, δ, and ε) (Calbiochem, San Diego, CA) was used at a concentration of 1 μM in an attempt to inhibit the conventional PKC pathway. Cells were either pretreated with the inhibitors for 1 h for western blot analysis or for 12 h for wound healing assays and then were treated with rWNT5A in the continuing presence of inhibitor or were treated with inhibitor alone for the indicated times. For the PKC activation studies, *phorbol*-12-myristate-*13*-*acetate* (PMA) (Sigma-Aldrich, St. Louis, MO) was used at 200 nM. For vehicle controls, the cells were treated with equivalent amounts of DMSO.

### Migration and invasion assays

For migration and invasion assays, 2 × 10^4^ NPC cells in 500 μl of serum-free DMEM were added to the cell culture inserts using an 8-μm microporous filter with a Matrigel coating (Becton Dickinson Labware, Bedford, MA) for the invasion assay or without Matrigel coating (migration assay). DMEM with 10% FBS was added to the bottom chamber. After 18 h of incubation, the cells on the lower surface of the filter were fixed and stained and then examined on microscopy. Cell counts in five random optical fields (×200 magnification) from triplicate filters were averaged.

### Immunoblotting and antibodies

Immunoblotting was performed as described previously [41, 45]. The sources of the primary antibodies (and their concentrations) were as follows: anti-PKC (anti-PKC alpha) (1:1,000), anti-phospho-pan-PKC (1:1,000), anti-β-catenin (1:1,000), anti-N-cadherin (1:1,000), anti-vimentin (1:1,000), anti-E-Cadherin (1:1,000), anti-ERK1/2 (1:1,000), anti-phospo-ERK1/2 (1:1,000), anti-JNK (1:1,000), anti-phospho-JNK (1:1,000), anti-AKT (1:1,000), anti-phospho-AKT (1:1,000), and anti-β-catenin (1:1,000) antibodies were purchased from Cell Signaling Technology (Danvers, MA); anti-WNT5A antibody (1:100) from R&D Biosystems (Minneapolis, MN); anti-Snail (1:200), anti-α-tubulin (1: 2,000), and anti-desmoplakin (1:1,000) antibodies from Abcam Inc (Cambridge, MA); anti-fibronectin (1:500) antibody from BD Biosciences (San Jose, CA); anti-lamin B1 (1:1,000) from Proteintech Group (Wuhan, China). To evaluate the nuclear and cytosolic fractions of β-catenin, a nuclear-cytosol extraction kit (FDbio Science, China) was used. Lamin B1 and α-tubulin were used as loading controls for the nuclear and cytoplasmic proteins, respectively.

### Human tissue samples

To compare the mRNA expression levels of *WNT5A* among different stages of NPC development, forceps biopsies of non-cancerous nasopharyngeal mucosa and primary NPCs were obtained at the Department of Nasopharyngeal Carcinoma, Sun Yat-sen University Cancer Center (SYSUCC). Ultrasound-guided core-needle biopsies of metastatic NPC tissues in cervical lymph nodes and metastatic NPC in the liver, both of which were later confirmed, were collected at the Department of Ultrasonography, SYSUCC. All of the tissues were collected with the patients' consent. Half of each forceps biopsy tissue was frozen immediately with liquid nitrogen, and the other half was processed for routine histological analysis. NPC tissue microarray (TMA) analyses were performed as previously described [7, 9]. Briefly, 220 cases of NPC with sufficient follow-up data were qualified for analyses after immunohistochemical (IHC) staining for WNT5A and vimentin. Another cohort of paraffin-embedded pulmonary NPC metastases tissues were also retrieved from the Department of Pathology, SYSUCC.

### Immunohistochemistry and histological evaluation

Immunohistochemical staining of WNT5A and vimentin was performed automatically using Discovery XT System from Ventana Medical Systems (Tucson, Ariz) according to the manufacturer's instructions as described previously [46]. Briefly, tissue sections were incubated with mouse anti-human WNT5A (dil: 1:5000, Cat. No.: ab86720, Abcam Inc., Cambridge, MA) or incubated with rabbit anti-vimentin (dil 1:200, clone V2009, Biomeda, Foster City). The reactions were visualized by secondary antibodies labeled with 3,30-diaminobenzidine (brown staining). The immunoreactions were evaluated independently by two pathologists. Cytoplasmic and membranous staining intensity were categorized: absent staining as 0, weak as 1, moderate as 2, and strong as 3. The percentage of stained cells was categorized as no staining = 0, 1–10% of stained cells = 1, 11–50% = 2, 51–80% = 3, and 81–100% = 4. The immunoreactive score for each tissue was calculated by multiplying the score of the staining intensity by that of the percentage of positive cells.

### Reverse transcription (RT)-PCR and quantitative real-time RT-PCR analysis

Total cellular and tissue RNA was extracted using the High Pure RNA Kit (Roche Applied Science, Penzberg, Germany). For RT-PCR, the total RNA was quantified spectrophotometrically, and equal amounts (1 μg) were transcribed into cDNA according to the manufacturer's protocol (Roche Applied Science, Penzberg, Germany). The following primers were used to amplify *WNT5A:* sense primer, 5′-CTTCGCCCAGGTTGTAATTGAAGC-3′; and antisense primer: 5′-CTGCCAAAAACAGAGGTGTTATCC-3′. Glyceraldehyde-3-phosphate dehydrogenase (*GAPDH*) was amplified as an internal control using the sense primer 5′-ACCACAGTCCATGCCATCAC-3′ and the antisense primer 5′-TCCACCACCCTGTTGCTGTA-3′. The appropriate size of the PCR products was confirmed by agarose gel electrophoresis.

In addition, mRNA levels from tissues were quantified by real-time RT-PCR analysis. The *WNT5A* and *GAPDH* primers were used to amplify 200 ng cDNA using the 7500 Fast Real-Time PCR system (Applied Biosystems, Inc., Rotkreuz, Switzerland) and the Power SYBR Green Master Mix (Takara Biotechnology, DaLian, China). The following primers were used for *WNT5A* and *GAPDH* in the real-time PCR assay:

*WNT5A* forward, 5′-AGCCCAGGAGTTGCTTTG-3′;
*WNT5A* reverse: 5′-TTCTGACATCTGAACAGGGTTA-3′;
*GAPDH* forward: 5′-GGAAGGTGAAGGTCGGAGTC-3′; and
*GAPDH* reverse: 5′-TGGGTGGAATCATATTGGAACA-3′.

Relative mRNA levels were quantified using the comparative Ct method. A melting curve analysis was performed for each amplicon to verify the specificity of each amplification step. The expression level of the WNT5A gene was normalized to the GAPDH level in each sample. The normalized fold expression levels of the WNT5A gene in different cancerous tissues were calculated in comparison to the WNT5A expression levels in the non-cancerous nasopharyngeal (NP) mucosa using the 2^−ΔΔCT^ calculation method according to the manufacturer's protocol (Perkin-Elmer).

### Flow cytometry

For the analysis of the cell surface markers, the cells in the exponential growth phase were counted, plated into a six-well plate at a 3×10^5^ cells/well, and cultured with RPMI 1640 complete medium for 24 hrs to allow settling. The cells were harvested and washed twice with PBS. The cells were labeled with a phycoerythrin (PE)-conjugated anti-CD44 antibody (BD Pharmingen^TM^, CA) and a ﬂuorescein isothiocyanate (FITC)-conjugated anti-CD24 antibody (BD Pharmingen^TM^, CA) Cells incubated without labeled antibody were used as negative controls. Cells were incubated with 1 μl of antibody per 1×10^6^ cells at room temperature for 30 min according to the manufacturer's instructions before detection on a flow cytometer. For side population analysis, suspending S26 cells at a density of 1 × 106 cells/mL were incubated with DNA-binding dye Hoechst 33342 (Sigma-Aldrich) at a concentration of 5 μg/mL for 90 minutes in dark and mixed every 15 minutes. Then the cells were washed twice with PBS, resuspended in PBS, kept at 4°C in the dark for flow cytometric analysis. For negative control, the cells were incubated with 10 μmol/L fumitremorgin C (FTC), which is a specific inhibitor for ABCG2 protein, for 5 minutes before the addition of the Hoechst dye. Flow cytometry was performed as described previously [46].

### Statistical analysis

Student's t-test was used to compare two independent groups of data. One-way analysis of variance (ANOVA) was used to analyze the significance among groups (non-cancerous nasopharyngeal mucosa, primary NPC, and metastatic NPC). The median IHC staining score was used as the cutoff value to divide the patients into low and high WNT5A or vimentin expression groups. Chi-squared tests were applied to analyze the relationship between WNT5A/vimentin expression and clinicopathological status. Kaplan-Meier survival curves were plotted, and log-rank tests were performed. The significance of several variables for survival was analyzed using the Cox regression model in a multivariate analysis. A P value < 0.05 was considered statistically significant in all cases.
